# Prognostic utility of the C-reactive protein–albumin–lymphocyte (CALLY) index in metastatic renal cell carcinoma

**DOI:** 10.1186/s12885-025-14767-9

**Published:** 2025-08-20

**Authors:** Güner Akgüner, Mustafa Altınbaş

**Affiliations:** Department of Medical Oncology, Etlik City Hospital, Ankara, Turkey

**Keywords:** Renal cell carcinoma, CALLY index, Albumin, Lymphocyte, CRP, Prognostic

## Abstract

**Background and objective:**

The CALLY index has been recognized as a prognostic biomarker across various malignancies; however, its prognostic utility in metastatic renal cell carcinoma (mRCC) has not been extensively characterized. This study aimed to elucidate the prognostic significance of the C-reactive protein–albumin–lymphocyte (CALLY) index in patients diagnosed with mRCC.

**Methods:**

In this retrospective multicenter study, patients with metastatic renal cell carcinoma were included. Demographic and disease-related data were collected. The CALLY index was calculated as follows: serum albumin level (g/dL) × absolute lymphocyte count (cells/µL)/C-reactive protein (CRP) (mg/dL) × 10^4^. The optimal cut-off value for the CALLY index was determined using maximal log-rank analysis. Overall survival and progression-free survival analyses were performed according to the cut-off value, and Cox regression analyses were conducted to identify factors affecting prognosis.

**Results:**

A total of 95 patients were included in the present study. The prognostic cut-off value for the CALLY index was determined as 0.12. The median overall survival (OS) was 11.1 months (95% CI 6.7–15.4) in the CALLY index < 0.12 group, compared to 45.7 months (95% CI 31.6–59.8) in the CALLY index ≥ 0.12 group (*p* < 0.001). Similarly, the median progression-free survival (PFS) was 7.1 months (95% CI 4.5–13.2) in the CALLY index < 0.12 group and 33.1 months (95% CI 17.2–48.1) in the CALLY index ≥ 0.12 group (*p* < 0.001). In Cox regression analysis, a CALLY index < 0.12 was identified as an independent prognostic factor for shorter OS (HR: 0.41, 95% CI 0.22–0.76, *p* = 0.004) and PFS (HR: 0.40, 95% CI 0.23–0.69, *p* = 0.001).

**Conclusion:**

The CALLY index was identified as an independent prognostic biomarker in metastatic renal cell carcinoma. Prospective large-scale studies are needed to confirm its clinical utility.

**Supplementary Information:**

The online version contains supplementary material available at 10.1186/s12885-025-14767-9.

## Introduction

Kidney cancer (KC) ranks as the 14th most frequently diagnosed malignancy worldwide, with an estimated 430,000 new cases annually, representing approximately 1.6% of all cancer-related deaths globally [1]. Renal cell carcinoma (RCC) accounts for nearly 80% of kidney cancers, with clear cell RCC being the most common histological subtype [2, 3]. The 5-year survival rate for localized RCC is relatively favorable, ranging between 88% and 93%; however, this rate declines markedly to approximately 15% in cases of advanced-stage disease [4]. In RCC, factors such as tumor stage, histological grade, local invasion, regional lymph node involvement, and the presence of distant metastases at the time of diagnosis are recognized as key prognostic indicators [5–8]. The International Metastatic Renal Cell Carcinoma Database Consortium (IMDC) model is widely used for prognostication in metastatic RCC, demonstrating significantly shorter overall survival in patients with intermediate- and poor-risk profiles compared to those in the favorable-risk group [9].

Systemic inflammation plays a well-established role in the progression and prognosis of renal cell carcinoma (RCC) [10]. Inflammation-related biomarkers, such as albumin and C-reactive protein (CRP), have been identified as prognostic indicators in RCC, with hypoalbuminemia and elevated CRP levels consistently associated with poor clinical outcomes [11–13]. The absolute lymphocyte count in peripheral blood is considered a surrogate marker of systemic immune and inflammatory status, which is closely linked to treatment response and survival. In RCC, baseline lymphopenia prior to treatment has been associated with shorter overall survival (OS) [14, 15].

Numerous immune and inflammatory markers—including the neutrophil-to-lymphocyte ratio (NLR), platelet-to-lymphocyte ratio (PLR), lymphocyte-to-CRP ratio, lymphocyte-to-monocyte ratio, and CRP-to-albumin ratio—have been shown to provide prognostic value in cancer patients [16–23]. The CRP–Albumin–Lymphocyte (CALLY) index is a composite prognostic marker incorporating C-reactive protein (CRP), albumin, and lymphocyte count, reflecting systemic inflammation, nutritional status, and immune function in cancer patients. It has demonstrated prognostic significance across several malignancies, including hepatocellular carcinoma, oral cavity cancer, breast cancer, and colorectal cancer, with lower CALLY scores consistently associated with worse clinical outcomes [24–28]. Although the CALLY index has been explored in various tumor types, evidence regarding its prognostic utility in metastatic renal cell carcinoma (mRCC) remains limited. This retrospective study aimed to evaluate the prognostic value of the CALLY index in patients with mRCC.

## Methods

### Patient selection and data collection

This retrospective multicenter study was conducted across two institutions: the Medical Oncology Departments of Ankara Etlik City Hospital and Dışkapı Yıldırım Beyazıt Training and Research Hospital. The study included patients treated between June 2014 and June 2024. Ethical approval was obtained from the Scientific Research Evaluation and Ethics Committee of Ankara Etlik City Hospital (Decision No: AEŞH-BADEK-2024-458; Date: June 5, 2024). The study was conducted in accordance with the principles of the Declaration of Helsinki.

Inclusion criteria were as follows: patients aged 18 years or older with histopathologically confirmed renal cell carcinoma (RCC) who either presented with metastatic disease at initial diagnosis or developed metastases during follow-up after an initial diagnosis of non-metastatic RCC; absence of pregnancy or breastfeeding; continuous clinical follow-up; and availability of sufficient data to calculate the CALLY index and assess disease status. Exclusion criteria included incomplete clinical or laboratory data and loss to follow-up during the study period. Medical records, clinical findings, and laboratory results of 110 patients were retrospectively reviewed, and 95 patients who met the inclusion criteria were ultimately included in the study.

Collected data encompassed demographic variables (age, sex, smoking history), Karnofsky performance status (KPS), sites of metastasis, history of nephrectomy, clinical TNM stage, metastatic burden categorized as oligometastatic (1–3 metastatic lesions) or polymetastatic (> 3 lesions), tumor histology (clear cell vs. non-clear cell), Fuhrman nuclear grade, laboratory parameters (hemoglobin, neutrophil count, lymphocyte count, calcium, lactate dehydrogenase [LDH], albumin), systemic treatments administered, IMDC risk classification at diagnosis, and CALLY index values calculated from blood tests obtained within one week prior to the initial evaluation and prior to the initiation of treatment or follow-up.

### Calculation of the Cally index score

The Cally index was calculated using the following formula [29]:

$$\begin{aligned}{\text{Cally Index }} & ={\text{ }}({\text{serum albumin level }}\left( {{\text{g}}/{\text{dL}}} \right) \\ & \quad \times {\text{absolute lymphocyte count }}({\text{cells}}/{\text{mL}}))/({\text{CRP level }} \\ & \quad \left( {{\text{mg}}/{\text{dL}}} \right) \times {\text{1}}{0^{\text{4}}}).\end{aligned}$$

### Study design and statistical method

Overall survival (OS) was defined as the time from diagnosis to death from any cause, whereas progression-free survival (PFS) was defined as the time from initiation of treatment to either documented disease progression or death from any cause. The primary endpoint of the study was OS, and the secondary endpoint was PFS. Time-dependent receiver operating characteristic (ROC) curve analysis was performed to assess the prognostic performance of the CALLY index at 12-month intervals using the ‘timeROC’ package in R software (version 4.5.0; accessed on 22 June 2025). Due to a limited number of patients under follow-up beyond 96 months, area under the curve (AUC) values were calculated from 12 to 96 months. Confidence intervals (CIs) for AUC estimates were derived using the bootstrap method with 1,000 resamples to evaluate the sensitivity and specificity of the CALLY index. The optimal cut-off value of the CALLY index for predicting OS was determined using maximal log-rank statistics via the ‘maxstat’ package. Based on this cut-off value, OS and PFS were compared between groups using the Kaplan–Meier method, and survival curves were generated using the ‘survival’ and ‘survminer’ packages in R.

Further analyses were performed using SPSS software (version 27.0; IBM Corp., Chicago, IL, USA) to evaluate clinicopathological factors with potential prognostic significance. Descriptive statistics were reported as mean ± standard deviation (SD), median (interquartile range, IQR), or frequency (n, %), as appropriate. Patients were stratified into two groups based on the CALLY index cut-off value. Categorical variables were compared using the Chi-square test or Fisher’s exact test, as applicable. Normality of continuous variables was assessed using the Shapiro–Wilk test. Normally distributed variables were compared between groups using the Independent Samples t-test, while non-normally distributed variables were compared using the Mann–Whitney U test. Univariate Cox proportional hazards regression analyses were conducted to identify potential prognostic factors for overall survival (OS) and progression-free survival (PFS). Multicollinearity among variables was evaluated using the Variance Inflation Factor (VIF), with all included variables showing VIF values between 1 and 2, indicating no significant collinearity. Variables found to be statistically significant in univariate analyses were included in multivariate Cox regression models. Survival times were expressed as median months with 95% confidence intervals (CIs). A two-sided p-value < 0.05 was considered statistically significant. Post-hoc power analysis was performed using G*Power version 3.1.9.4. Based on observed mortality rates in the CALLY < 0.12 (33/38) and ≥ 0.12 (30/57) groups, the calculated statistical power was 94% (actual α = 0.036), indicating sufficient power to detect clinically meaningful differences in OS between groups.

## Results

### Comparison of baseline characteristics between the high and low CALLY index groups

**Fig. 1:**
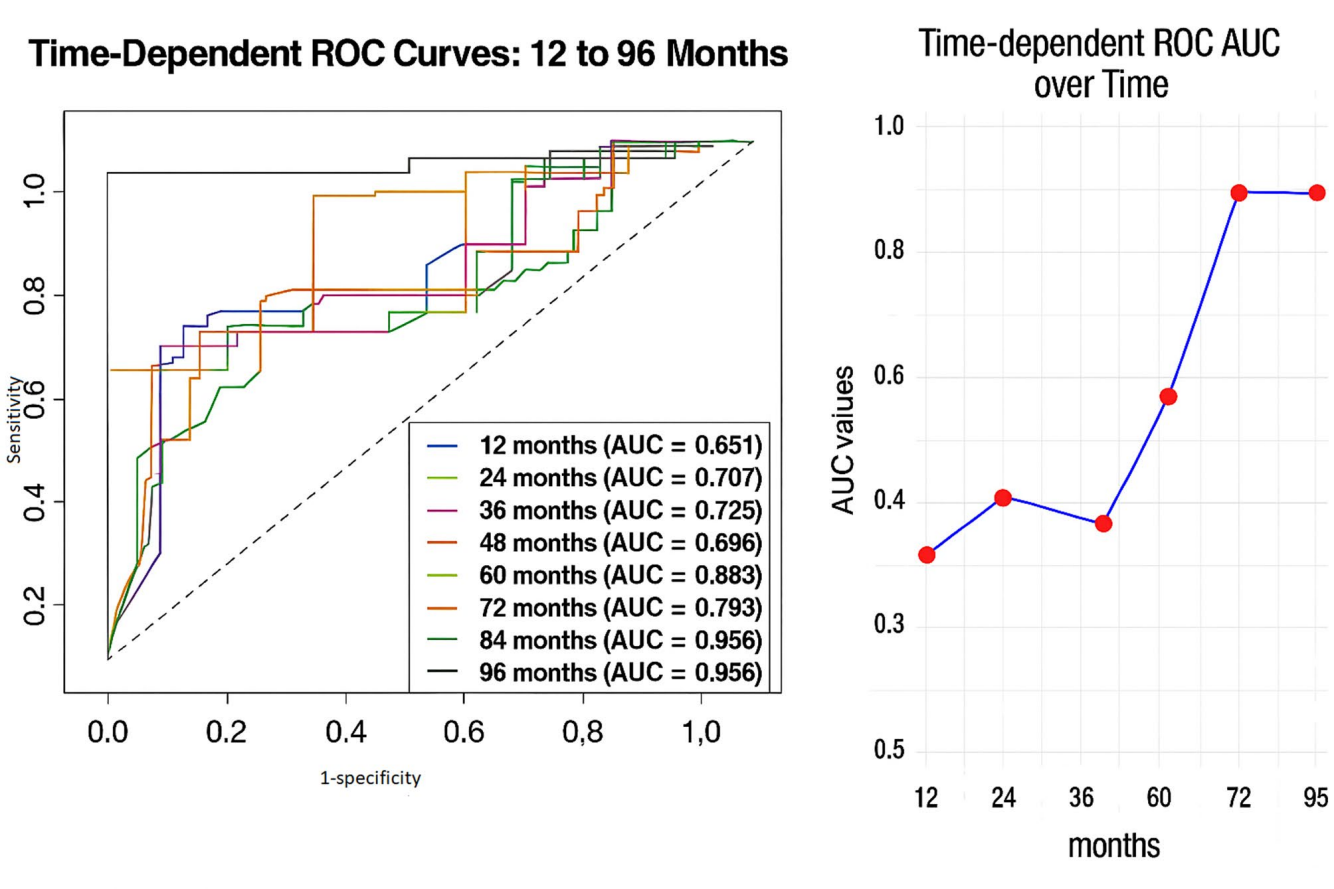
Time-dependent ROC curves based on CALLY Index and AUC values of the ROC curve across 12- to 96-month periods

Patient characteristics and laboratory results are summarized in Tables 1 and 2. Maximal log-rank analysis identified an optimal CALLY index cut-off value of 0.12 (log-rank *p* < 0.001). Using this cut-off, time-dependent receiver operating characteristic (ROC) curve analysis was performed at 12-month intervals from month 12 to month 96, yielding area under the curve (AUC) values of 0.651, 0.707, 0.725, 0.696, 0.685, 0.793, 0.956, and 0.956, respectively (Table 3; Fig. 1).

**Table 1:** Baseline clinical and laboratory features between two CALLY groups

	Cally Index	Mean (± SD)	*p*
Age	< 0.12	63.5 (± 11.1)	0.283
	≥ 0.12	60.8 (± 12.7)	
*Median + IQR*			
Albumin (g/dL)	< 0.12	3.2 + 0.6	**< 0.001**
	≥ 0.12	4.2 + 0.8	
Lymphocyte (10^3^/mm^3^)	< 0.12	0.895 + 0.865	**< 0.001**
	≥ 0.12	1.790 + 1.175	
LDH (U/L)	< 0.12	252.5 + 143.0	0.057
	≥ 0.12	223.0 + 89.0	
Platelet (10^3^/mm^3^)	< 0.12	2.640 + 1.625	0.185
	≥ 0.12	2.880 + 1.855	
Hemoglobin (g/dL)	< 0.12	10.85 + 2.40	**< 0.001**
	≥ 0.12	13.0 + 1.80	
Calcium (mg/dL)	< 0.12	8.9 + 1.3	**0.002**
	≥ 0.12	9.4 + 1.3	
Neutrophil (10^3^/mm^3^)	< 0.12	5.470 + 4.080	0.412
	≥ 0.12	5.200 + 2.530	
CRP (mg/dL)	< 0.12	6.5 + 6.4	**< 0.001**
	≥ 0.12	1.3 + 1.4	

Bold values indicate statistically significant results with *p* < 0.05

Patients with a CALLY index < 0.12 exhibited significantly lower serum albumin, lymphocyte count, hemoglobin, and calcium levels compared to those with a score ≥ 0.12 (*p* < 0.001, *p* < 0.001, *p* < 0.001, and *p* = 0.002, respectively). Conversely, C-reactive protein (CRP) levels were significantly higher in the CALLY < 0.12 group compared to the ≥ 0.12 group (*p* < 0.001) (Table 1).

Lymph node metastasis, liver metastasis, and poor-risk status according to the IMDC classification were more frequent among patients with a CALLY index < 0.12 than in those with scores ≥ 0.12 (*p* = 0.043, *p* = 0.040, and *p* = 0.003, respectively) (Table 2).

**Table 2:** Patient characteristics by CALLY groups and treatment lines

		Cally Index		*p*
		< 0.12	≥ 0.12	
Sex	Female	11 (28.9%)	17 (29.9%)	0.927
	Male	27 (71.1%)	40 (70.1%)	
Smoking	No	11 (28.9%)	15 (26.4%)	0.778
	Yes	27 (71.1%)	42 (73.6%)	
Oligometastatic/polymetastatic disease	Oligometastatic	3 (7.8%)	10 (17.6%)	0.180
	Polymetastatic	35 (92.2%)	47 (82.4%)	
Perineural invasion	No	16 (42.1%)	29 (50.9%)	0.402
	Yes	22 (57.9%)	28 (49.1%)	
Lymphovascular invasion	No	18 (47.4%)	35 (61.5%)	0.177
	Yes	20 (52.6%)	22 (38.5%)	
Bone metastasis	No	21 (55.3%)	29 (50.9%)	0.675
	Yes	17 (44.7%)	28 (49.1%)	
Brain metastasis	No	29 (76.3%)	50 (87.8%)	0.146
	Yes	9 (23.7%)	7 (12.2%)	
Lung metastasis	No	13 (34,2%)	25 (65.8%)	0.789
	Yes	18 (31,6%)	39 (68.4%)	
Lymph node metastasis	No	16 (42.1%)	22 (57.9%)	**0.043**
	Yes	36 (63.1%)	21 (36.9%)	
Liver metastasis	No	21 (55.3%)	43 (75.4%)	**0.040**
	Yes	17 (44.7%)	14 (24.6%)	
Nephrectomy	No	11 (28.9%)	20 (35.1%)	0.532
	Yes	27 (71.1%)	37 (64.9%)	
Histology	Non-clear cell	4 (10.5%)	5 (8.8%)	0.775
	Clear cell	34 (89.5%)	52 (91.2%)	
IMDC risk group	Intermediate	13 (34.2%)	37 (64.9%)	**0.003**
	Poor	25 (65.8%)	20 (35.1%)	
TNM stage	Stage 1	1 (2.6%)	0 (0.0%)	0.537
	Stage 2	3 (7.9%)	8 (14.0%)	
	Stage 3	12 (31.6%)	16 (28.1%)	
	Stage 4	22 (57.9%)	33 (57.9%)	
Fuhrman grade	Grade 2	14 (36.8%)	22 (38.6%)	0.073
	Grade 3	14 (36.8%)	30 (52.6%)	
	Grade 4	10 (26.3%)	5 (8.8%)	
KPS	< 80	21 (48.8%)	22 (51.2%)	0.110
	≥ 80	17 (32.7%)	35 (67.3%)	

	Treatment agents and lines	*n*	%	
First line	Cabozantinib	8	8.4	
	Nivolumab + Cabozantinib	4	4.2	
	Pazopanib	26	27.4	
	Pembrolizumab	1	1.1	
	Sunitinib	45	47.4	
	Best supportive care	11	11.5	
Second-line	Axitinib	3	3.2	
	Everolimus	8	8.4	
	Nivolumab	17	17.9	
	Pazopanib	2	2.1	
Third-line	Axitinib	5	5.3	
	Cabozantinib	1	1.1	
	Everolimus	1	1.1	
	Cabozantinib	3	3.2	
	Nivolumab	2	2.1	
Fourth-line	Axitinib	2	2.1	
	Cabozantinib	1	1.1	
	Everolimus	1	1.1	
	Sunitinib	1	1.1	
Fifth-line	Cabozantinib	1	1.1	

Bold values indicate statistically significant results with *p* < 0.05

**Table 3:** Area under the curve (AUC) with 95% confidence interval for the CALLY index at 12 months

Months	AUC	95% CI
12	0.65	0.53–0.76
24	0.71	0.58–0.82
36	0.73	0.58–0.85
48	0.70	0.55–0.83
60	0.68	0.50–0.85
72	0.79	0.58–0.97
84	0.96	0.89–1.00
96	0.96	0.89–1.00

### Survival analyses of patients

**Fig. 2:**
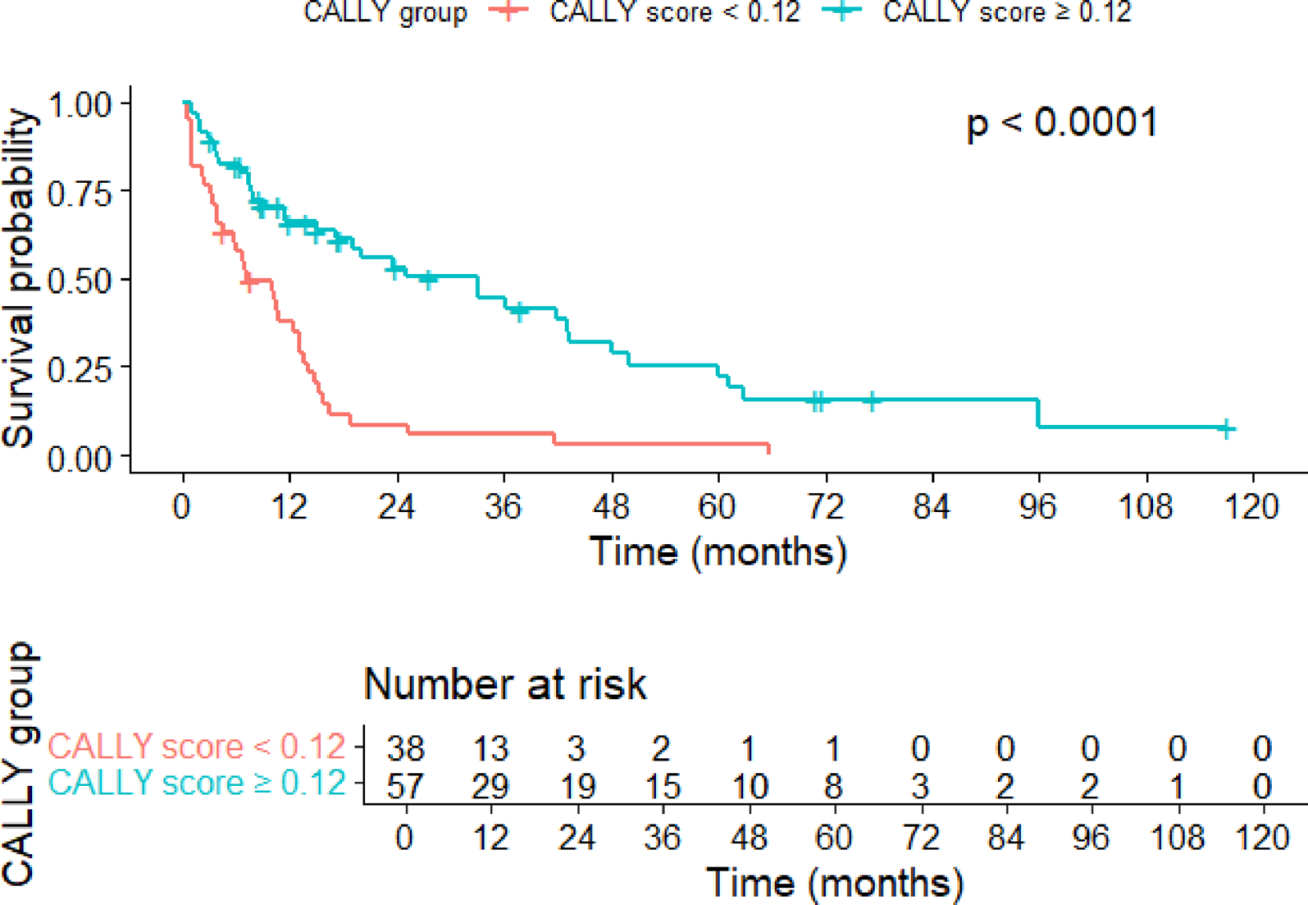
Progression-free survival (PFS) curve stratified by CALLY Index groups

**Fig. 3:**
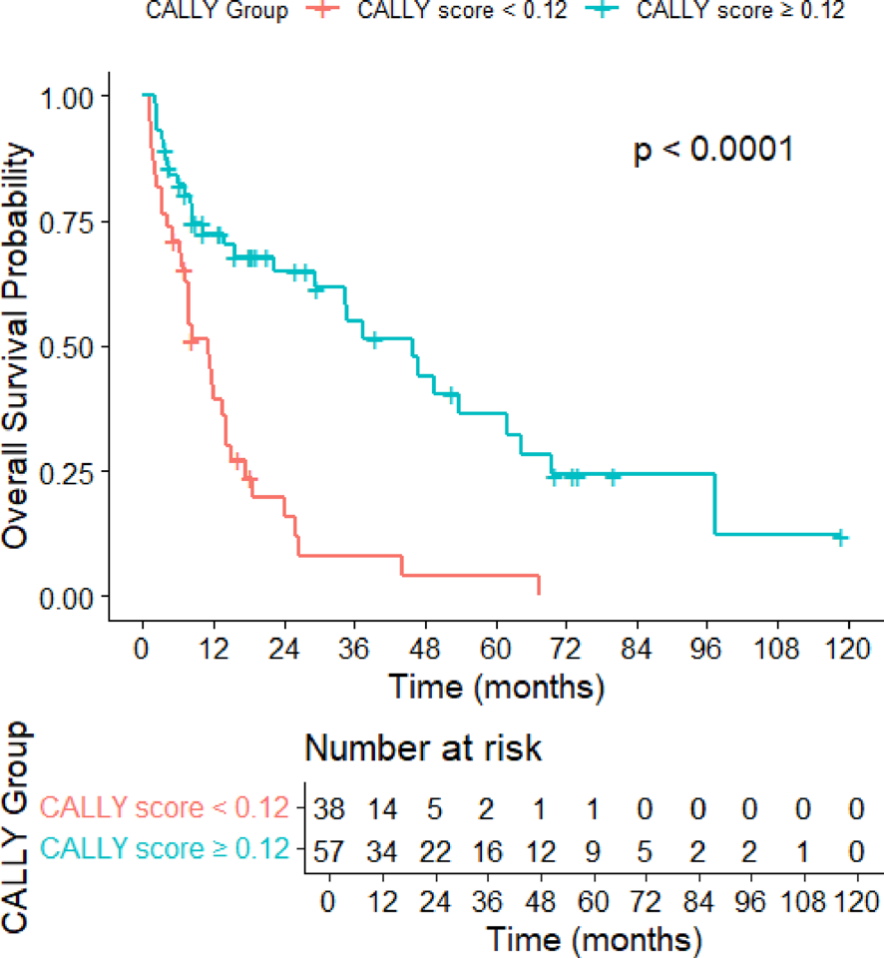
Overall survival (OS) curve stratified by CALLY Index groups

The median overall survival (OS) was 17.5 months (95% CI 8.0–27.0), and the median progression-free survival (PFS) was 13.5 months (95% CI 10.4–19.1). Survival analyses stratified by treatment agents, regardless of therapy line, are summarized in Table 4 (Supplementary File 1). Due to the limited number of patients treated with pembrolizumab and cabozantinib plus nivolumab, survival analyses for these agents were not conducted. Kaplan–Meier survival curves and log-rank tests revealed a median progression-free survival (mPFS) of 7.1 months (95% CI 4.5–13.2) in the CALLY index < 0.12 group compared to 33.1 months (95% CI 17.2–48.1) in the ≥ 0.12 group (*p* < 0.001) (Fig. 2). Similarly, median overall survival (mOS) was 11.1 months (95% CI 6.7–15.4) for patients with a CALLY index < 0.12 and 45.7 months (95% CI 31.6–59.8) for those with ≥ 0.12 (*p* < 0.001) (Fig. 3).

**Table 4:** Survival analysis according to CALLY groups in all patients and by treatment agents

	CALLY Index	Median OS (95% CI)	*p*	Median PFS (95% CI)	*p*
Patient	< 0.12	11.1 (6.7–15.4)	< 0.001	7.1 (4.5–13.2)	< 0.001
	≥ 0.12	45.7 (31.6–59.8)		33.1 (17.2–48.1)	
	Overall	17.5 (8.0–27.0)		13.5 (10.4–19.1)	

		Median OS		Median PFS	
Sunitinib	< 0.12	14.0	**< 0.001**	13.0	**< 0.001**
	≥ 0.12	37.5		36.1	
Pazopanib	< 0.12	7.8	**0.044**	7.0	0.233
	≥ 0.12	46.7		33.1	
Cabozantinib	< 0.12	67.3	0.774	15.8	0.689
	≥ 0.12	65.7		33.1	
Nivolumab	< 0.12	25.8	0.154	15.8	0.447
	≥ 0.12	61.9		24.3	
Axitinib	< 0.12	26.4	**0.008**	15.8	**< 0.001**
	≥ 0.12	61.9		60.1	
Everolimus	< 0.12	18.6	**< 0.001**	15.2	**< 0.001**
	≥ 0.12	49.5		41.8	

Bold values indicate statistically significant results with *p* < 0.05

### Other prognostic analyses

Multivariate analysis identified low Karnofsky performance status (< 80%) (hazard ratio [HR]: 0.48, 95% confidence interval [CI]: 0.28–0.81, *p* = 0.006) and CALLY index < 0.12 (HR: 0.40, 95% CI 0.23–0.69, *p* = 0.001) as independent risk factors for shorter progression-free survival (PFS).

Additionally, low Karnofsky performance status (< 80%) (HR: 0.38, 95% CI 0.21–0.67, *p* = 0.001), polymetastatic disease (HR: 8.77, 95% CI 1.15–66.9, *p* = 0.036), and CALLY index < 0.12 (HR: 0.41, 95% CI 0.22–0.76, *p* = 0.004) were identified as independent predictors of shorter overall survival (OS) (Table 5).

**Table 5:** Univariate and multivariate Cox regression analyses of prognostic factors for OS and PFS

		Median PFS (month)	Univariate analysis for PFS		Multivariate analysis for PFS		Median OS (month)	Univariate analysis for OS		Multivariate analysis for OS	
			HR (95% CI)	*p*	HR (95% CI)	*p*		HR (95% CI)	*p*	HR (95% CI)	*p*
Age	< 62	17.2	1.57, 0.96–2.54	0.065			25.7	1.37, 0.82–2.28	0.215		
	≥ 62	11.4					13.6				
KPS (%)	< 80	7.0	**0.35**,** 0.21–0.56**	**< 0.001**	**0.48**,** 0.28–0.81**	**0.006**	7.8	**0.26**,** 0.15–0.45**	**< 0.001**	**0.38**,** 0.21–0.67**	**0.001**
	≥ 80	20.0					44.2				
Fuhrman grade	Grade 2	15.8	**1.52**,** 1.08–2.13**	**0.016**			26.3	**1.72**,** 1.20–2.49**	**0.003**		
	Grade 3	15.0					15.7				
	Grade 4	6.0					7.8				
TNM stage			1.24, 0.87–1.76	0.205				1.36, 0.92–2.01	0.110		
Smoking	No	12.5	0.93, 0.55–1.56	0.792			13.6	0.77, 0.45–1.31	0.344		
	Yes	14.7					24.0				
Oligometastatic/polymetastatic disease	Oligometastatic	49.9	**3.70**,** 1.48–9.28**	**0.005**			-	**16.2**,** 2.25–118.0**	**0.006**	**8.77**,** 1.15–66.9**	**0.036**
	Polymetastatic	11.4					14.0				
Perineural invasion	No	14.1	1.15, 0.72–1.84	0.547			26.3	1.42, 0.85–2.36	0.176		
	Yes	12.5					13.6				
Lymphovascular invasion	No	15.8	1.56, 0.98–2.49	0.058			26.3	**1.72**,** 1.04–2.83**	**0.032**		
	Yes	10.3					11.4				
Nephrectomy	No	6.6	**0.58**,** 0.36–0.95**	**0.038**			7.6	**0.53**,** 0.31–0.89**	**0.018**		
	Yes	15.0					24.0				
Histology	Non-clear cell	8.8	0.51, 0.25–1.03	0.085			10.2	**0.42**,** 0.20–0.87**	**0.020**		
	Clear cell	14.7					24.0				
Bone metastasis	No	14.1	1.02, 0.64–1.63	0.905			17.5	1.06, 0.64–1.74	0.813		
	Yes	11.4					18.6				
Brain metastasis	No	14.7	1.81, 1.02–3.22	0.054			22.3	**2.31**,** 1.28–4.17**	**0.005**		
	Yes	6.0					7.8				
Lung metastasis	No	13.2	1.21, 0.73–2.03	0.445			15.1	1.18, 0.68–2.05	0.547		
	Yes	13.5					17.5				
Liver metastasis	No	15.2	1.45, 0.89–2.38	0.141			29.3	**1.67**,** 1.00-2.78**	**0.049**		
	Yes	8.8					10.2				
Lymph node metastasis	No	14.7	1.27, 0.78–2.07	0.322			29.3	1.31, 0.78–2.21	0.305		
	Yes	13.0					14.1				
Cally Index	< 0.12	7.1	**0.32**,** 0.20–0.53**	**< 0.001**	**0.40**,** 0.23–0.69**	**0.001**	11,1	**0.29**,** 0.17–0.50**	**< 0.001**	**0.41**,** 0.22–0.76**	**0.004**
	≥ 0.12	33.1					45.7				
IMDC risk group	Intermediate	19.1	**2.24**,** 1.39–3.62**	**0.001**			49,5	**3.26**,** 1.90–5.59**	**< 0.001**		
	Poor	10.1					11.1				

Bold values indicate statistically significant results with *p* < 0.05

## Discussion

Although previous studies have demonstrated the prognostic utility of the CRP-albumin-lymphocyte (CALLY) index in various cancer types, data regarding its role in metastatic renal cell carcinoma (mRCC) remain limited. Our findings suggest that the CALLY index serves as a valuable prognostic biomarker for predicting both overall survival (OS) and progression-free survival (PFS) in patients with mRCC.

The CALLY index is a non-invasive composite marker derived from peripheral blood parameters—C-reactive protein (CRP), albumin, and lymphocyte count—that collectively reflect systemic inflammation, nutritional status, and immune function. Hypoalbuminemia is well established as an indicator of poor nutritional status and disease severity, correlating with adverse outcomes across multiple malignancies [30–32]. Elevated CRP, as an inflammatory marker, has been consistently linked to poorer prognosis in cancer patients [33, 34]. Lymphocytes are pivotal in anti-tumor immunity, and low pre-treatment lymphocyte counts have been associated with unfavorable outcomes in solid tumors [35, 36]. The increasing use of immune checkpoint inhibitors in mRCC underscores the critical role of tumor-immune interactions, further emphasizing the relevance of lymphocyte dynamics in this context. In our cohort, patients with a CALLY index < 0.12 exhibited significantly lower serum albumin and higher CRP levels, indicating impaired nutritional status and heightened systemic inflammation. These biochemical alterations likely contribute to the observed reductions in both OS and PFS within this subgroup. Although the central role of lymphocytes in orchestrating anti-tumor immune responses within the tumor microenvironment is well recognized, the peripheral lymphopenia observed in patients with low CALLY scores warrants further investigation to clarify the underlying biological mechanisms.

Several studies have corroborated the prognostic significance of the CALLY index across diverse malignancies. Iida et al. reported significantly lower 5-year OS and recurrence-free survival in hepatocellular carcinoma patients with a CALLY index < 5 compared to those with higher scores [24]. Similarly, Feng et al. demonstrated that higher preoperative CALLY index values were associated with improved 5-year cancer-specific survival in esophageal cancer [37]. In colorectal cancer, a low CALLY index was linked to increased mortality risk, outperforming other immunonutritional markers such as the modified Glasgow Prognostic Score, neutrophil-to-lymphocyte ratio, systemic immune-inflammation index, and platelet-to-lymphocyte ratio [26]. Additional evidence from studies on pancreatic and gastric cancers also supports the association between lower CALLY indices and poorer survival outcomes [38, 39]. Hirata et al. identified the CALLY index as a significant prognostic marker in RCC patients post-nephrectomy, where lower scores correlated with shorter PFS and higher postoperative recurrence rates [40].

The heterogeneity in optimal CALLY cut-off values across cancer types may reflect tumor-specific differences in the interplay of immune, nutritional, and inflammatory factors. Nevertheless, consistently across these studies, lower CALLY index values correspond with worse prognosis. Our findings, demonstrating shorter PFS and OS among patients with a CALLY index < 0.12, align with this body of evidence. Notably, the higher proportion of IMDC poor-risk patients within the low CALLY group further underscores its prognostic relevance. Moreover, in multivariate analyses, the CALLY index emerged as an independent predictor of both PFS and OS, outperforming the IMDC risk score—a novel and clinically relevant observation.

This study has several limitations. Its retrospective design and relatively small sample size may limit generalizability. Variations in treatment regimens and the administration of multiple therapy lines, without adjustment for treatment line in survival analyses, might confound outcomes. The small number of patients receiving pembrolizumab or cabozantinib plus nivolumab precluded survival analyses for these regimens, representing another constraint. The notably high area under the curve (AUC) values observed at 84 and 96 months could be influenced by reduced patient numbers at these time points, raising the possibility of overfitting. Additionally, while the CALLY index benefits from its simplicity and reliance on routine blood tests, it may be affected by various patient-related factors unrelated to cancer status, which could impact its accuracy. Importantly, the CALLY cut-off value identified in this study has not undergone external validation. Future research should focus on prospective, multicenter studies to externally validate the prognostic utility of the CALLY index in mRCC, ideally incorporating subgroup analyses by treatment regimens. Integration of the CALLY index into artificial intelligence–based multivariable prognostic models may enhance its clinical applicability, potentially serving as a decision support tool in personalized cancer care. Finally, the inherent susceptibility of peripheral blood–based markers to confounding factors warrants cautious interpretation of findings and underscores the need for comprehensive biomarker panels.

## Conclusion

This study evaluated the prognostic utility of the CALLY index in patients with metastatic renal cell carcinoma (mRCC). Our findings demonstrate that the CALLY index serves as a significant predictor of both overall survival and progression-free survival in this patient population. Further large-scale, prospective validation studies are warranted to confirm and reinforce the clinical applicability of the CALLY index in mRCC management.

## Supplementary Information

Supplementary Material 1: Kaplan-Meier survival (OS and PFS) curves by treatment agents. Contains 12 Kaplan-Meier survival plots stratified by treatment agents. These figures were referenced in the Results section and included as supplementary material due to space constraints.

## Data Availability

The data used and/or analyzed during the current study are available from the corresponding author on reasonable request.
