# Supplementary material for: Prognostic utility of the C-reactive protein–albumin–lymphocyte (CALLY) index in metastatic renal cell carcinoma
Source: BMC Cancer. 2025 Aug 20;25:1347. doi: 10.1186/s12885-025-14767-9 (PMC12369225; doi:10.1186/s12885-025-14767-9)
Supplement: Supplementary file 1 — Supplementary Material 1: Kaplan-Meier survival (OS and PFS) curves by treatment agents. Contains 12 Kaplan-Meier survival plots stratified by treatment agents. These figures were referenced in the Results section and included as supplementary material due to space constraints. [file 12885_2025_14767_MOESM1_ESM.pdf]

1. Kaplan–Meier Overall Survival (OS) Curves Stratified by CALLY Groups Across Treatment Agents

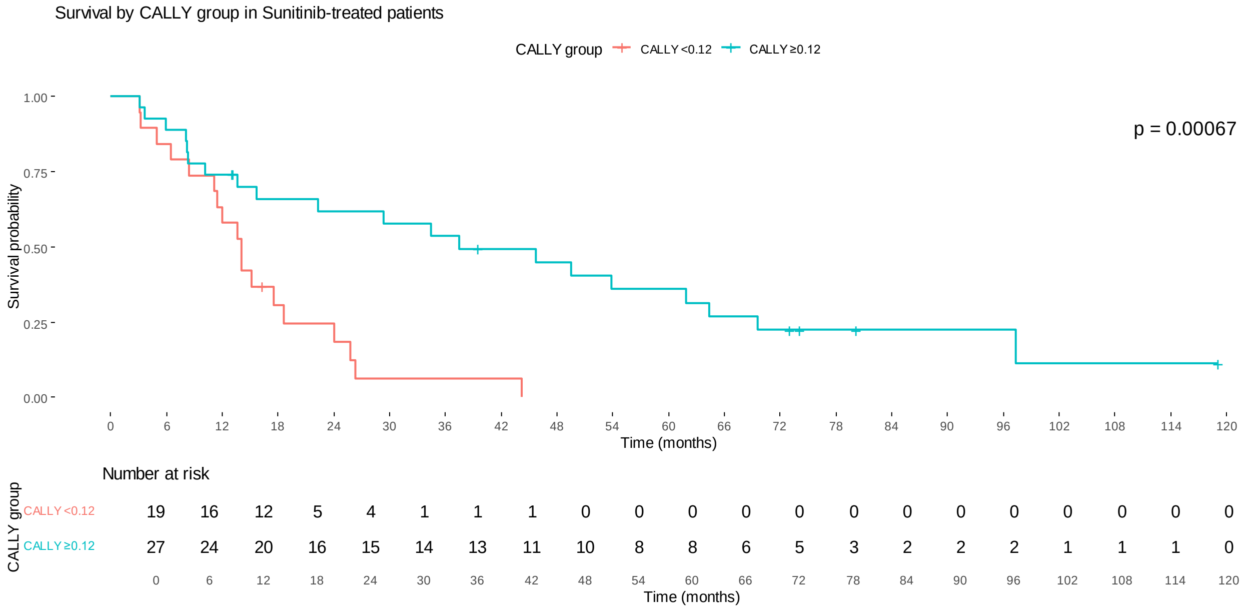

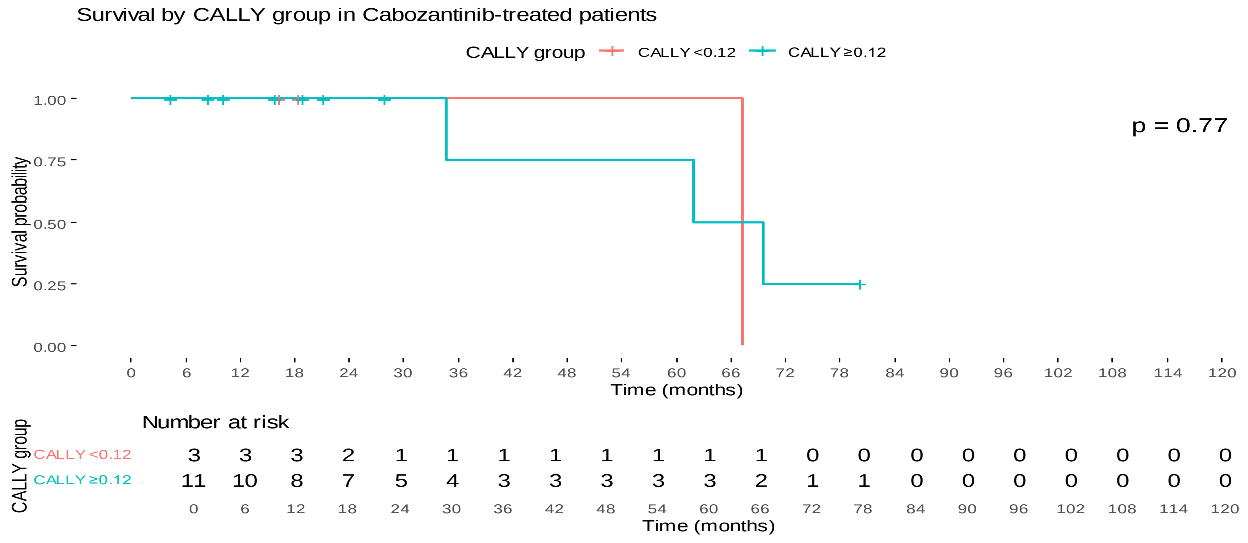

Survival by CALLY group in Pazopanib-treated patients

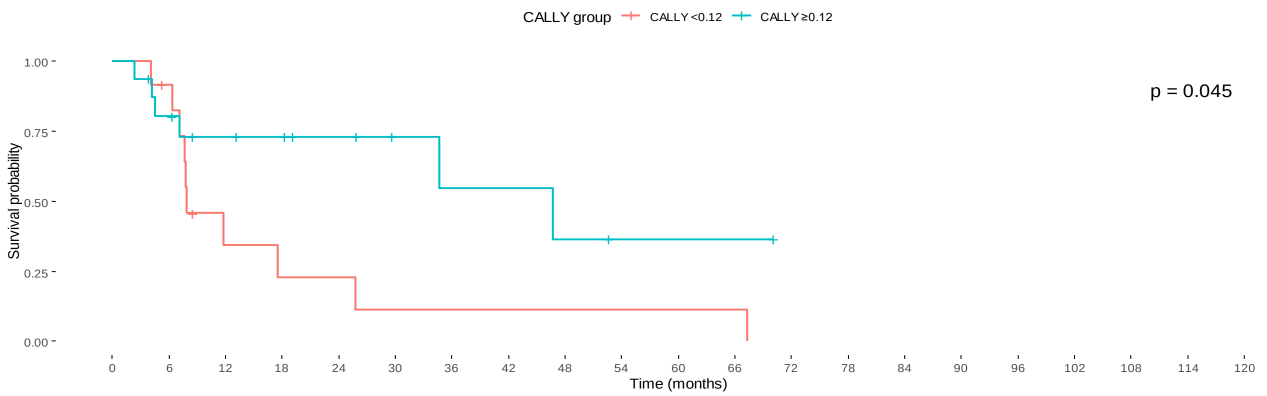

Number at risk

CALLY group	0	6	12	18	24	30	36	42	48	54	60	66	72	78	84	90	96	102	108	114	120
CALLY <0.12	12	10	3	2	2	1	1	1	1	1	1	1	0	0	0	0	0	0	0	0	0
CALLY ≥0.12	16	12	9	8	6	4	3	3	2	1	1	1	0	0	0	0	0	0	0	0	0

Time (months)

Survival by CALLY group in Nivolumab-treated patients

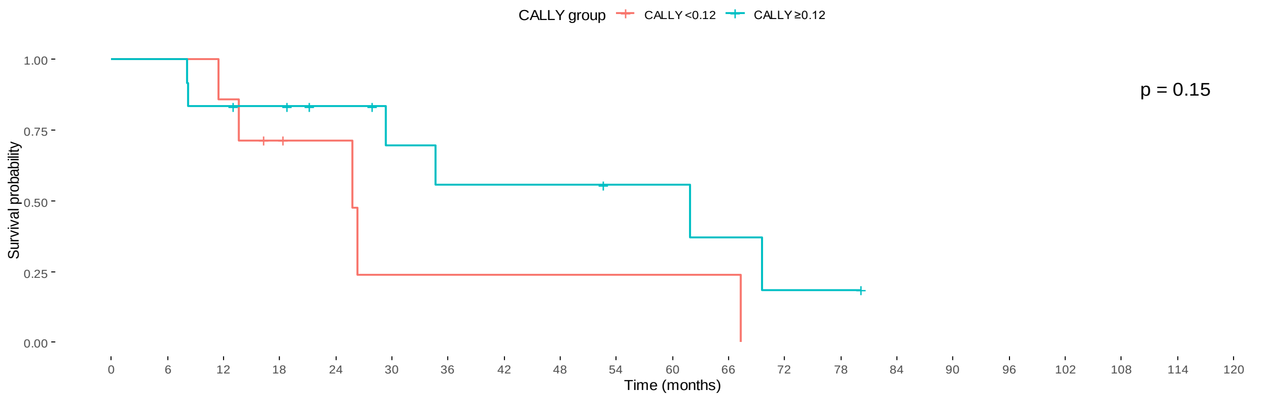

Number at risk

CALLY group	0	6	12	18	24	30	36	42	48	54	60	66	72	78	84	90	96	102	108	114	120
CALLY <0.12	7	7	6	4	3	1	1	1	1	1	1	1	0	0	0	0	0	0	0	0	0
CALLY ≥0.12	12	12	10	9	7	5	4	4	4	3	3	2	1	1	0	0	0	0	0	0	0

Time (months)

Survival by CALLY group in Axitinib-treated patients

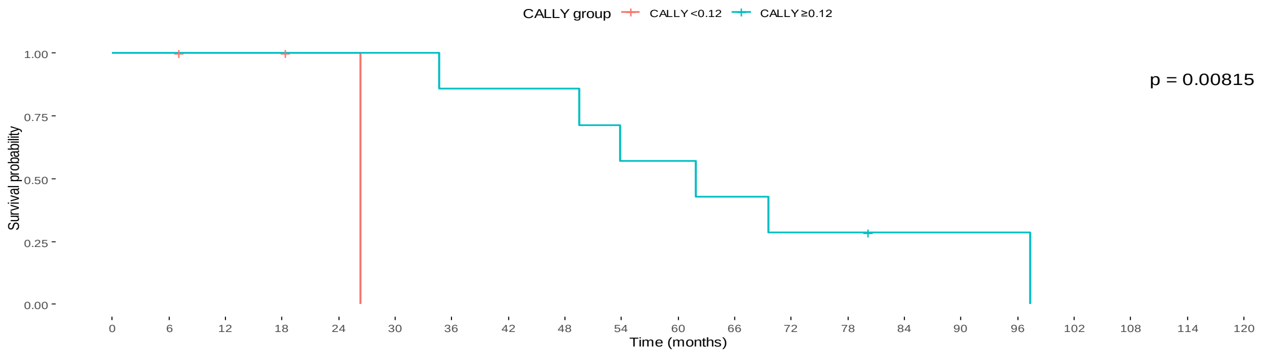

Number at risk

CALLY group	0	6	12	18	24	30	36	42	48	54	60	66	72	78	84	90	96	102	108	114	120
CALLY <0.12	3	3	2	2	1	0	0	0	0	0	0	0	0	0	0	0	0	0	0	0	0
CALLY ≥0.12	7	7	7	7	7	7	6	6	6	4	4	3	2	2	1	1	1	0	0	0	0

Time (months)

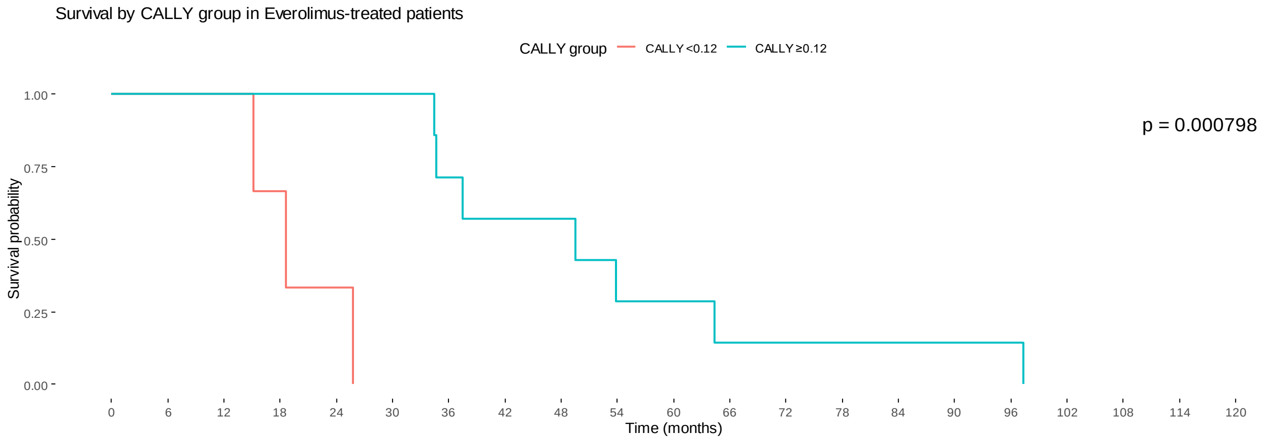

Number at risk

CALLY group	0	6	12	18	24	30	36	42	48	54	60	66	72	78	84	90	96	102	108	114	120
CALLY <0.12	3	3	3	2	1	0	0	0	0	0	0	0	0	0	0	0	0	0	0	0	0
CALLY ≥0.12	7	7	7	7	7	7	5	4	4	2	2	1	1	1	1	1	1	0	0	0	0

Time (months)

2. Kaplan–Meier Progression-free Survival (PFS) Curves Stratified by CALLY Groups Across Treatment Agents

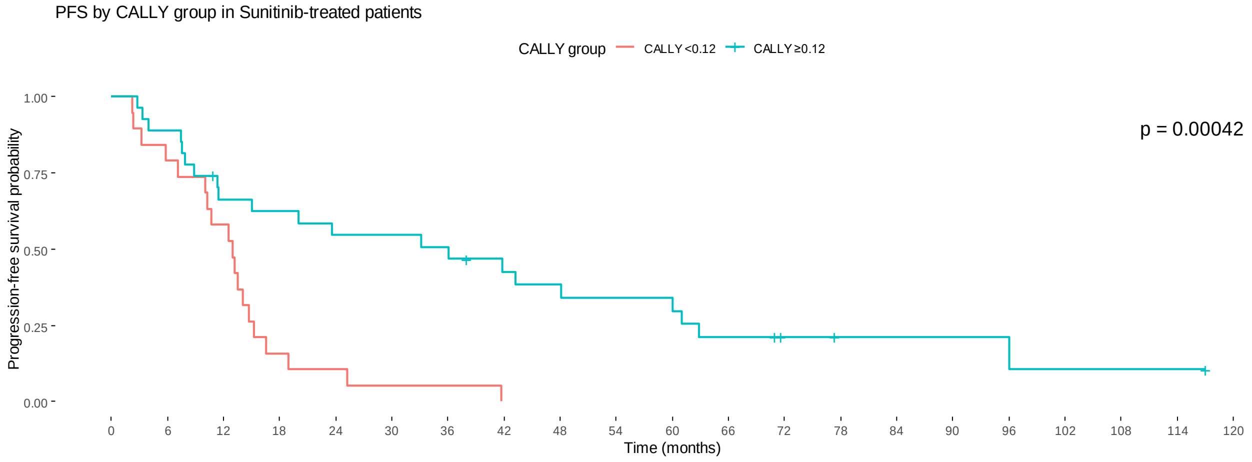

Number at risk

CALLY group	0	6	12	18	24	30	36	42	48	54	60	66	72	78	84	90	96	102	108	114	120
CALLY <0.12	19	15	11	3	2	1	1	0	0	0	0	0	0	0	0	0	0	0	0	0	0
CALLY ≥0.12	27	24	17	16	14	14	13	10	9	8	8	5	3	2	2	2	2	1	1	1	0

Time (months)

PFS by CALLY group in Cabozantinib-treated patients

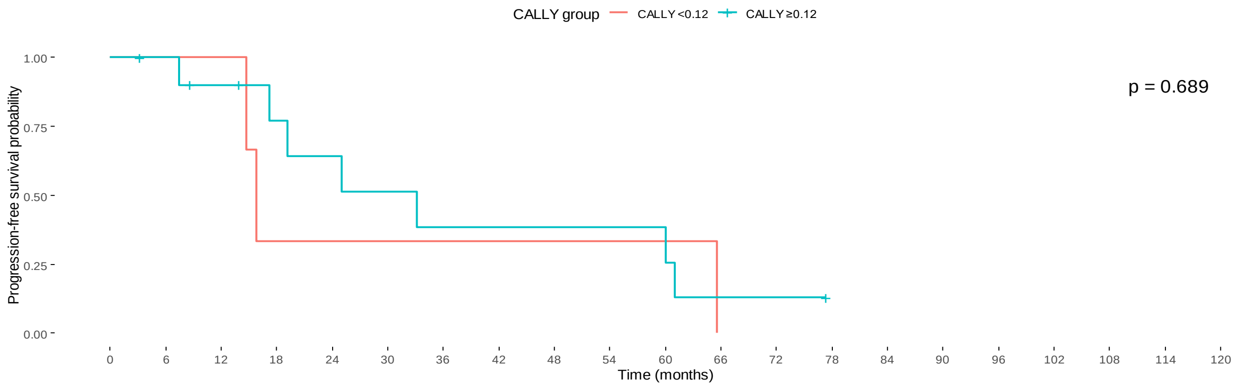

Number at risk

CALLY group	0	6	12	18	24	30	36	42	48	54	60	66	72	78	84	90	96	102	108	114	120
CALLY <0.12	3	3	3	1	1	1	1	1	1	1	1	0	0	0	0	0	0	0	0	0	0
CALLY ≥0.12	11	10	8	6	5	4	3	3	3	3	3	1	1	0	0	0	0	0	0	0	0

Time (months)

PFS by CALLY group in Pazopanib-treated patients

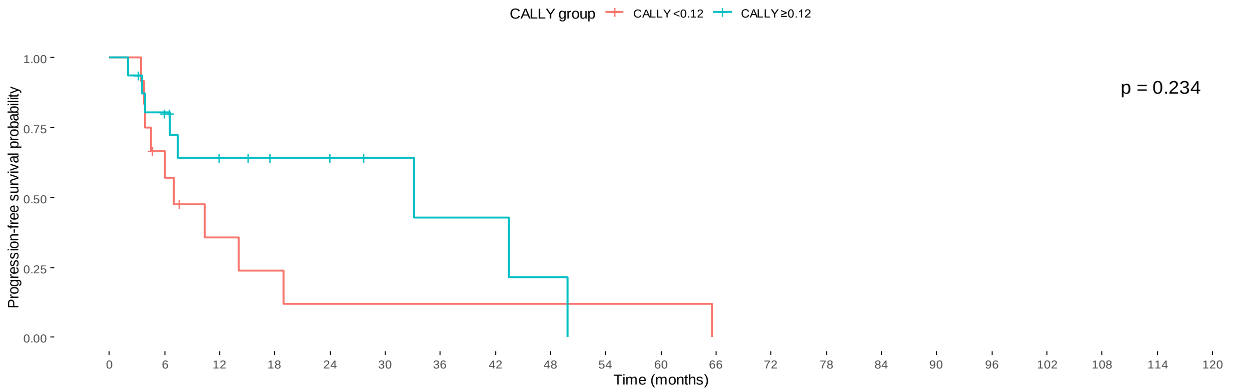

Number at risk

CALLY group	0	6	12	18	24	30	36	42	48	54	60	66	72	78	84	90	96	102	108	114	120
CALLY <0.12	12	7	3	2	1	1	1	1	1	1	1	0	0	0	0	0	0	0	0	0	0
CALLY ≥0.12	16	11	7	5	4	3	2	2	1	0	0	0	0	0	0	0	0	0	0	0	0

Time (months)

PFS by CALLY group in Nivolumab-treated patients

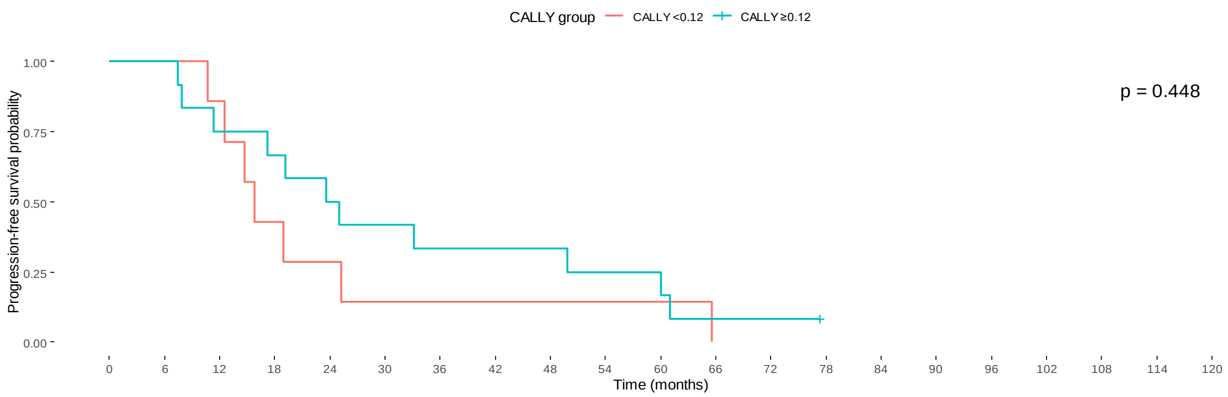

Number at risk

CALLY group	0	6	12	18	24	30	36	42	48	54	60	66	72	78	84	90	96	102	108	114	120
CALLY<0.12	7	7	6	3	2	1	1	1	1	1	1	0	0	0	0	0	0	0	0	0	0
CALLY≥0.12	12	12	9	8	6	5	4	4	4	3	3	1	1	0	0	0	0	0	0	0	0

Time (months)

PFS by CALLY group in Axitinib-treated patients

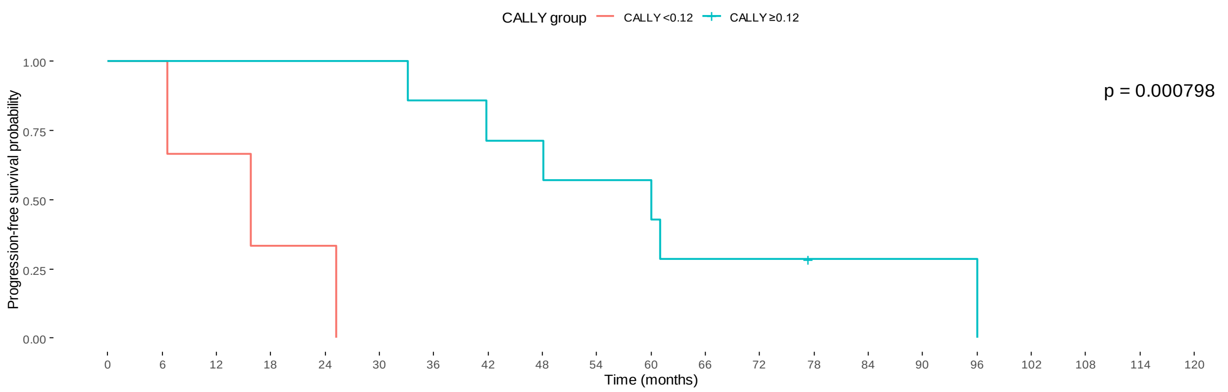

Number at risk

CALLY group	0	6	12	18	24	30	36	42	48	54	60	66	72	78	84	90	96	102	108	114	120
CALLY<0.12	3	3	2	1	1	0	0	0	0	0	0	0	0	0	0	0	0	0	0	0	0
CALLY≥0.12	7	7	7	7	7	7	6	5	5	4	4	2	2	1	1	1	1	0	0	0	0

Time (months)

PFS by CALLY group in Everolimus-treated patients

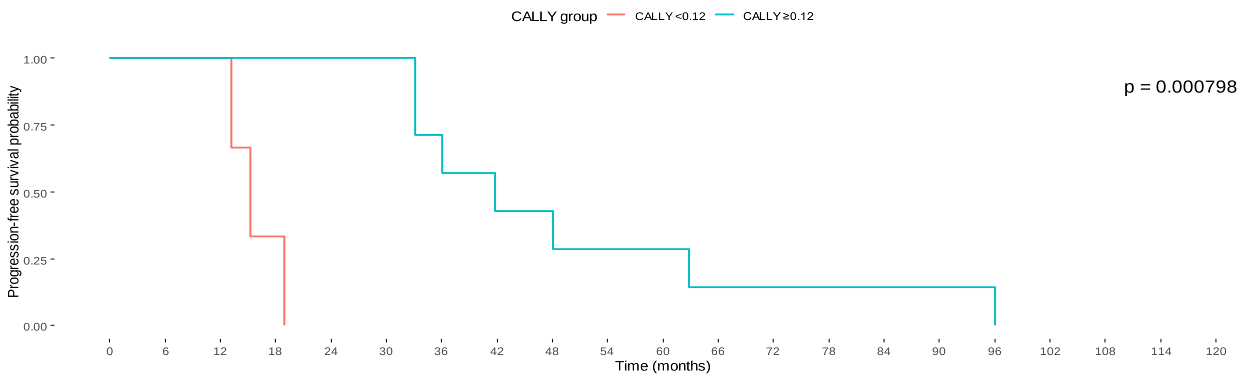

Number at risk

CALLY group	0	6	12	18	24	30	36	42	48	54	60	66	72	78	84	90	96	102	108	114	120
CALLY<0.12	3	3	3	1	0	0	0	0	0	0	0	0	0	0	0	0	0	0	0	0	0
CALLY≥0.12	7	7	7	7	7	7	5	3	3	2	2	1	1	1	1	1	1	0	0	0	0

Time (months)
